# Mediation Role of C-Reactive Protein on the Association between Smoking Quantity and Type 2 Diabetes in Current Chinese Smokers

**DOI:** 10.1155/2014/171538

**Published:** 2014-07-03

**Authors:** Dan Feng, Tao Liu, Hui Wang, Emma Karp, Wenhua Ling, Wei-Qing Chen

**Affiliations:** ^1^Department of Preventive Medicine, School of Public Health, Sun Yat-Sen University, Guangzhou 510080, China; ^2^Guangdong Provincial Key Laboratory of Food, Nutrition and Health, School of Public Health, Sun Yat-Sen University, Guangzhou 510080, China; ^3^Department of Biostatistics and Epidemiology, School of Public Health, Sun Yat-Sen University, Northern Campus, 74 Zhongshan Road 2, Guangzhou 510080, China; ^4^Guangdong Provincial Institute of Public Health, Guangdong Provincial Center for Disease Control and Prevention, Guangzhou 511430, China; ^5^Guangdong Key Laboratory of Molecular Epidemiology, School of Public Health, Guangdong Pharmaceutical University, Guangzhou 510300, China; ^6^Department of Clinical Nutrition, School of Public Health, Sun Yat-Sen University, Guangzhou 510080, China

## Abstract

*Objective*. Previous studies have indicated that cigarette smokers are more likely to develop type 2 diabetes and that both smoking and type 2 diabetes are associated with C-reactive protein (CRP). This study examined whether CRP mediates the association between smoking quantity and type 2 diabetes. *Methods*. Nine hundred and eighty-four current Chinese smokers were selected from a community-based chronic disease survey conducted in Guangzhou and Zhuhai. Type 2 diabetes was defined according to the WHO 1999 criteria. CRP was measured with flow cytometry. Binary logistic regression was performed to assess the mediation. *Results*. A positive association was observed between smoking quantity and type 2 diabetes (*P* < 0.05). After controlling for potential confounders, daily cigarette consumption was significantly associated with higher CRP levels. Current smokers with type 2 diabetes had higher CRP levels than smokers without type 2 diabetes. The association between the smoking quantity and type 2 diabetes was mediated by CRP, which accounted for 50.77% of the association. *Conclusions*. This study provides further evidence that smoking quantity is positively associated with type 2 diabetes and suggests that the association between smoking and type 2 diabetes might be mediated by CRP.

## 1. Introduction

Type 2 diabetes occurs commonly worldwide and its development is associated with many factors, including lifestyle factors [1–3]. Increasing evidence has indicated that cigarette smoking is an independent risk factor for type 2 diabetes [4–6]. Compared with nonsmokers, both current and former smokers are at significantly greater risk of developing type 2 diabetes [5, 7]. Experts have observed a clear dose-response relationship between smoking quantity and the incidence of type 2 diabetes [4, 5]. Despite this evidence, the mechanism by which cigarette smoking causes type 2 diabetes remains unclear.

We hypothesised that inflammation may play a mediation role in the process by which cigarette smoking causes type 2 diabetes. Levels of C-reactive protein (CRP), a sensitive marker of systemic inflammation, were found to be elevated in individuals with features of metabolic syndrome and insulin resistance [8–10]. Higher CRP levels were also shown to be positively associated with an increased risk of type 2 diabetes [11–13]. Some of the heavy metals and tobacco glycoprotein components in cigarettes are proinflammatory and cigarette smoking was proven to be positively correlated with inflammation [14]. For example, the National Health and Nutrition Examination Survey III found a positive dose relationship between cigarette smoking and levels of CRP and fibrinogen [15]. The Multinational Monitoring of Trends and Determinants in Cardiovascular Disease study discovered a higher CRP baseline in former smokers than nonsmokers 20 years after smoking cessation [16]. Type 2 diabetes is considered an inflammatory disease and inflammation may play an important role in the pathogenesis of type 2 diabetes [17]. Taylor et al. showed that an inflammatory marker of fibrinogen partly mediated the effect of cigarette smoking on depression [18] and we found that CRP mediated the association between smoking quantity and hypertension [19]. Whether CRP also mediates the association between smoking quantity and type 2 diabetes is still unresolved and is the subject of this study.

## 2. Methods

### 2.1. Subjects

A community-based chronic disease survey was conducted in Guangzhou and Zhuhai, China, from July 2006 to June 2007 [20]. Of the 7293 residents aged 20 years or over who were randomly selected using a stratified multistage sampling method to take part in the survey, 1440 were smokers. We excluded 96 of the current smokers for whom there were no available blood samples and 360 former smokers, as current and former smokers represent different inflammatory conditions [21]. The final sample consisted of 984 current smokers. This study was approved by the Ethics Committees of Sun Yat-Sen University in Guangzhou of China and written informed consent was obtained from all of the selected participants.

### 2.2. Data Collection

All of the participants were interviewed by well-trained medical students or clinicians. Their sociodemographic characteristics of age, gender, income, education level, marital status, occupation, and smoking behaviour were collected using a structured questionnaire. The survey was conducted at local health care centres. A vein blood sample was obtained at the same time from each subject to measure the serum levels of inflammatory markers.

### 2.3. Measurement and Definition of Smoking Behaviour

Those who had smoked more than 100 cigarettes in their lifetimes and had smoked at least one cigarette daily for the six months prior to the survey were defined as current smokers [22]. Every current smoker was asked to report the average number of cigarettes consumed per day in their latest smoking period. According to the number of cigarettes smoked daily, the current smokers were grouped into light smokers (smoking ≤10 cigarettes per day), moderate smokers (smoking 11–20 cigarettes per day), and heavy smokers (smoking >20 cigarettes per day) [23].

### 2.4. Definition of Alcohol Consumption and Physical Activity

Those who had consumed alcohol at least three times a week for more than six months were defined as drinkers. The rest of the participants were defined as nondrinkers [24]. Subjects who undertook recreational physical activities at any intensity for 30 minutes at least three times a week were regarded as performing regular physical activity. If not, they were regarded as performing irregular physical activity [25].

### 2.5. Measurement of Inflammation

Based on the manufacturer's instructions, we measured serum CRP levels using flow cytometric (bead-based) multiplex assays (BMS8288FF and BMS8213FF, eBioscience, USA) on the BD FACSCalibur instruments (BD Biosciences). Fluorescence-labelled or streptavidin-labelled detection antibodies were bound to a specific cytokine-capture antibody complex on a bead set coated with a specific capture antibody. We measured the cytokine levels in biological liquid samples with fluorogenic emissions detected using a flow cytometric analysis. We collected the data using the Cell Quest software (BD Biosciences) and analysed it using FlowCytomix Pro (eBioscience).

### 2.6. Diagnostic Criteria for Type 2 Diabetes

The occurrence of type 2 diabetes was defined by self-reported history of type 2 diabetes, confirmed by the use of insulin or oral hypoglycemic agents, or a newly diagnosed case of type 2 diabetes according to the WHO diagnostic criteria for diabetes (fasting glucose ≥ 7.0 mmol/L or 2 h postprandial glucose ≥ 11.1 mmol/L) [20]. Cases of type 1 diabetes were not included.

### 2.7. Statistical Analysis

Means ± standard deviation (SD) were calculated for the continuous variables. The categorical variables were expressed as the percentage of subjects with the respective attribute. Chi-square tests were used to examine the association of type 2 diabetes (0 = no and 1 = yes) with the sociodemographic characteristics and CRP levels, which were divided into four quartile groups (1 = 0–25th, 2 = 26–50th, 3 = 51–75th, and 4 = 76–100th percentile). When the conditions for the chi-square test were not satisfied, Fisher's exact test was used. After adjusting for potential confounders, several logistic regression models were used to test the association between smoking quantity (1 ≤10, 2 = 11–20, and 3 >20 cigarettes/day) and type 2 diabetes.

The mediating effect of CRP on the relationship between the number of cigarettes smoked per day and type 2 diabetes was tested using a series of hierarchical regressions (binary logistic regression models or ordinal logistic regression models), again adjusting for potential confounders. According to Baron and Kenny [26], mediation is demonstrated when the main independent variable (i.e., smoking) is significantly associated with the main dependent variable (i.e., type 2 diabetes); the independent variable (i.e., smoking) is significantly associated with the mediator variable (i.e., CRP); and the mediator variable (i.e., CRP) is significantly associated with the dependent variable (i.e., type 2 diabetes) when the independent variable (i.e., smoking) is controlled for. The size of the mediation effect was evaluated by *ab*/(*ab* + *c*′) [27], where *a* is the coefficient relating the independent variable to the mediator, *b* is the coefficient relating the mediator to the dependent variable while adjusting for the independent variable, and *c*′ is the coefficient relating the independent variable to the dependent variable while adjusting for the mediator. The potential confounding factors were age, gender, occupation, education, monthly income, a family history of diabetes, alcohol consumption, and physical exercise.

All of the *P* values were two-sided and statistical significance was assessed at *P* = 0.05. The analysis was conducted using the SPSS 13.0 software package (SPSS Inc., Chicago, IL, USA).

## 3. Results

### 3.1. Social-Demographic Characteristics

The social-demographic characteristics of the 984 Chinese current smokers are shown in Table 1. There were significant differences in the ages, daily cigarette consumptions, family histories of diabetes, and serum CRP levels of the current smokers with and without type 2 diabetes.

### 3.2. Association of CRP with Daily Cigarette Consumption and Type 2 Diabetes

Table 2 presents the results of the ordinal logistic regression analysis of the correlation between the serum CRP level and the number of cigarettes smoked per day, after adjusting for the potential confounding factors. Table 2 shows that the number of cigarettes consumed per day was significantly and positively associated with the serum CRP level (*P* < 0.05).

After adjusting for the potential confounding factors, current smokers with type 2 diabetes had significantly higher serum levels of CRP than smokers without type 2 diabetes (Table 3).

### 3.3. Mediation Effect of CRP on the Association between Daily Cigarette Consumption and Type 2 Diabetes

Before and after controlling for age, gender, occupation, education, monthly income, family history of diabetes, alcohol consumption, and physical exercise, the results showed that smokers consuming over 20 cigarettes per day were at significantly greater risk of type 2 diabetes. Those smoking 11–20 cigarettes per day were at no greater risk of type 2 diabetes than those smoking ≤10 cigarettes per day (Model 1 and Model 2 in Table 4). After CRP was added into Model 2, the association between the number of cigarettes consumed per day and type 2 diabetes lessened and was no longer significant (Model 2a in Table 4), but the association between CRP and type 2 diabetes was still significant (Model 2a in Table 4).

**Figure 1:**
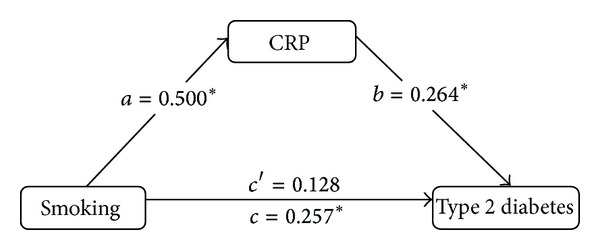
The mediation of CRP on the association between daily smoking quantity and type 2 diabetes.

Figure 1 illustrates the mediation effect and shows that CRP mediated the association between the daily smoking quantity and type 2 diabetes. The effect can be expressed as (0.500 × 0.264)/[(0.500 × 0.264) + 0.128] = 50.77%.

## 4. Discussion

CRP is an acute-phase reactant produced mainly by the hepatocytes in response to inflammatory stimuli. It has been shown to be a sensitive nonspecific biomarker of systematic inflammation [28]. The circulating value of CRP reflects ongoing inflammation and/or tissue damage [28] and is associated with cardiovascular disease, type 2 diabetes, smoking, and a sedentary lifestyle [29]. This study found a significant positive correlation between the serum CRP level, daily smoking quantity, and type 2 diabetes after adjusting for the potential confounders of age, gender, occupation, education, monthly income, family history of diabetes, alcohol consumption, and physical exercise. Further analysis showed that CRP mediated the association between the daily smoking quantity and type 2 diabetes, accounting for 50.77% of the association. These findings support the hypothesis that inflammation may play a mediating role in smoking causing type 2 diabetes.

### 4.1. Associations between CRP, Cigarette Smoking, and Type 2 Diabetes

Studies on cigarette smoking and CRP in men have consistently shown that current smokers have a higher level of CRP than exsmokers and nonsmokers [15, 16, 30, 31]. Some studies revealed a dose-response relationship between the smoking pack-year and the number of cigarettes smoked daily with elevated CRP levels [15, 16, 30]. Similarly, we found that the number of cigarettes smoked per day was significantly related to elevated serum CRP levels. However, Helmersson et al. did not find a significant difference between the levels of serum CRP in current smokers, exsmokers, and nonsmokers [21] and Fröhlich et al. did not discover a positive association between serum CRP levels and smoking status, the number of cigarettes smoked per day, or duration of the smoking period in women [16]. Although these reports on the relationship between smoking and the serum CRP level were inconsistent, they generally suggest that cigarette smoking may increase the secretion of CRP in the body.

It is widely recognised that CRP is an independent risk factor for type 2 diabetes. A recent meta-analysis involving 22 cohorts comprising 40,735 subjects and 5,753 cases indicated that elevated CRP levels were significantly associated with an increased risk of type 2 diabetes [32]. Similarly, our study found that higher serum CRP levels were significantly related to type 2 diabetes, after adjusting for potential confounders.

### 4.2. Association between Cigarette Smoking and Type 2 Diabetes and Its Mediation by CRP

There is strong epidemiological evidence that cigarette smoking is an independent risk factor for type 2 diabetes [4–6]. Recently, Willi et al. systematically reviewed 25 cohort studies on the association between smoking and the incidence of type 2 diabetes and found an adjusted relative risk of 1.44 (95% CI = 1.31–1.58) and a dose-response relationship between smoking and type 2 diabetes. More specifically, they found that those with a current heavy smoking habit (>20 cigarettes/day) were at greater risk for developing type 2 diabetes than those who smoked less [4]. We similarly found a dose-response association between the number of cigarettes smoked daily and type 2 diabetes and that heavy smokers were at significantly greater risk of developing type 2 diabetes than light smokers. CRP mediated this association between the smoking quantity and type 2 diabetes, accounting for 50.77% of the association. It should be noted that Taylor et al. indicated that an inflammatory marker of fibrinogen partly mediated the effect of cigarette smoking on depression [18] and that our previous study found that CRP mediated the association between smoking quantity and hypertension [19].

There are several possible mechanisms by which CRP may mediate the effect of cigarette smoking on the development of type 2 diabetes. Cigarette smoking may cause abdominal obesity (an accumulation of visceral fat mass in the abdominal area), which may induce the production and secretion of CRP in the hepatocytes and endothelial cells. It was observed that current smokers had a larger waist-to-hip ratio (an indicator of abdominal obesity) than nonsmokers [33] and that abdominal adiposity was significantly associated with elevated CRP levels [34]. CRP may impair *β*-cells' secretory function and interfere with early insulin signal transduction and glucose transport-1 effects [35]. CRP may detrimentally affect the vascular wall by reducing nitric oxide bioavailability and inducing endothelial dysfunction [36], which may impair insulin endocytosis in endothelial cells [37]. CRP may also inhibit the expression of peroxisome proliferator-activated receptors and precede insulin resistance. Any or all of these possible mechanisms may contribute to the development of type 2 diabetes [38].

These findings suggest that smoking and inflammation are two nodes in a web of risk factors for type 2 diabetes. The discovered mediating effects may offer new opportunities for clinical interventions to reduce the risk associated with one variable and make it easier to intervene at the other nodes in the pathogenic network of type 2 diabetes. For example, increased inflammation may contribute to type 2 diabetes induced by smoking. Diminishing inflammation through anti-inflammatory medications may therefore be clinically useful in type 2 diabetes patients who wish to quit smoking and are having difficulty. Conversely, smokers who become type 2 diabetes patients may be more resistant to antidiabetic medications than nonsmokers. Interventions that reduce inflammation may be useful additions to the treatment of type 2 diabetes in this context.

### 4.3. Limitations

The following limitations should be pointed out. This was a cross-sectional study and a causal relationship between cigarettes, CRP, and type 2 diabetes cannot be concluded. A longitudinal study design is needed to assess whether cigarette smoking is a factor that directly induces CRP and indirectly causes type 2 diabetes. The data were retrospectively collected using a self-administrated questionnaire, so information bias could not be avoided. The type 2 diabetes patients included in the survey were prevalent cases and data on the diabetes patients' antidiabetic medications were not collected. We therefore could not exclude the possible influence of antidiabetic medications on the association of CRP with cigarette smoking and type 2 diabetes. However, previous studies have found that some antidiabetic medications affect the inflammatory response [39, 40]. Although we made a great effort to increase the response rate, a low response rate could not be avoided in the healthy population. Less healthy individuals may be oversampled in our study, which may have led to a selection bias.

## 5. Conclusions

Heavy cigarette smoking (consuming >20 cigarettes/day) significantly increased the risk of developing type 2 diabetes. The observed relationship between the daily smoking quantity and type 2 diabetes may have been mediated by CRP.

## Comments

Previous studies have indicated that smoking is an independent risk factor for type 2 diabetes and that both smoking and type 2 diabetes are associated with inflammation. Whether smoking induces type 2 diabetes through inflammation remains unclear. Our study confirmed the previous findings of associations between smoking, CRP, and type 2 diabetes and further found that the association between smoking and type 2 diabetes was partially mediated by CRP. These findings may provide a novel insight into the mechanism by which smoking causes type 2 diabetes and in the therapeutic implications of smoking-related type 2 diabetes.

## Figures and Tables

**Table 1:** Comparison of social-demographic characteristics between type 2 diabetes and non-type 2 diabetes current smokers.

	Type 2 diabetes		*χ* ^2^	*P*
	Controls *N* (%)	Cases *N* (%)		
Age (years)				
<30	44 (5.0)	0 (0.0)	37.00	<0.001
30~39	103 (11.7)	3 (3.0)		
40~49	220 (24.9)	26 (25.7)		
50~59	297 (33.6)	25 (24.8)		
60~69	180 (20.4)	32 (31.7)		
≥70	39 (4.4)	15 (14.9)		
Gender				
Male	824 (93.3)	93 (92.1)	0.22	0.640
Female	59 (6.7)	8 (7.9)		
Occupation				
Person in charge	109 (12.3)	8 (7.9)	9.88	0.079
Technician	129 (14.6)	11 (10.9)		
Service personnel	122 (13.8)	19 (18.8)		
Operator	204 (23.1)	21 (20.8)		
Retired personnel	206 (23.3)	34 (33.7)		
Jobless	113 (12.8)	8 (7.9)		
Education				
Elementary school or lower	175 (19.8)	21 (20.8)	0.81	0.847
Junior middle school	310 (35.1)	31 (30.7)		
Senior middle school or vocational secondary school	288 (32.6)	35 (34.7)		
College or above	110 (12.5)	14 (13.9)		
Family monthly income (yuan)				
<1000	117 (13.3)	14 (13.9)	2.23	0.693
1000~2999	301 (34.1)	29 (28.7)		
3000~4999	242 (27.4)	27 (26.7)		
≥5000	132 (14.9)	20 (19.8)		
Do not know or refuse to answer	91 (10.3)	11 (10.9)		
Alcohol consumption				
No	579 (65.6)	63 (62.4)	0.939	0.625
Yes	261 (29.6)	31 (30.7)		
Former drinker	43 (4.9)	7 (6.9)		
Exercise				
No	225 (25.5)	28 (27.7)	0.24	0.625
Yes	658 (74.5)	73 (72.3)		
Daily cigarette consumption (cigarettes per day)				
≤10	393 (44.5)	45 (44.6)	6.88	0.032
11~20	417 (47.2)	40 (39.6)		
>20	73 (8.3)	16 (15.8)		
Family history of diabetes				
No	762 (86.3)	78 (77.2)	6.00	0.015
Yes	121 (13.7)	23 (22.8)		

	M ± SD	M ± SD	*Z* ^#^	*P*

Inflammatory marker				
CRP (*μ*g/mL)	4.5 ± 5.9	5.2 ± 4.9	2.79	0.005

^#^Mann-Whitney nonparametric test.

Type 2 DM: type 2 diabetes.

**Table 2:** The association between number of cigarettes smoked per day and CRP in 984 Chinese current smokers (OR; 95% CI)^#^.

Inflammatory marker	Number of cigarettes smoked per day		
	≤10	11~20	>20
CRP	1	1.47∗ (1.16–1.87)	3.29∗ (2.13–5.08)

^#^The adjusted OR was calculated by ordinal logistic regression models with adjustment for age, gender, occupation, education, family monthly income, family history of diabetes, alcohol consumption, and exercise.

*P < 0.05.

**Table 3:** The association between CRP and type 2 diabetes in 984 Chinese current smokers (OR; 95% CI)^#^.

	CRP			
	Quartile 1	Quartile 2	Quartile 3	Quartile 4
Type 2 diabetes	1	0.85 (0.42–1.74)	1.64 (0.87–3.06)	1.99∗ (1.08–3.67)

^#^The adjusted OR was calculated by binary logistic regression models with adjustment for age, gender, occupation, education, family monthly income, family history of diabetes, alcohol consumption, and exercise.

*P < 0.05.

**Table 4:** The association between number of cigarettes smoked per day and type 2 diabetes and the mediation by CRP in 984 Chinese current smokers (OR; 95% CI)^#^.

Model	Adjustments	Number of cigarettes smoked per day			Inflammatory markers				Nagelkerke *R* ^2^ (%)
		≤10	11~20	>20	Quartile 1	Quartile 2	Quartile 3	Quartile 4	
1	Smoking	1	0.84 (0.54–1.31)	1.91∗ (1.03–3.57)	—	—	—	—	1.10
2	Model 1 + confounders of age, and so forth	1	0.94 (0.60–1.49)	2.12∗ (1.10–4.14)	—	—	—	—	2.90
2a	Model 2 + CRP	1	0.84 (0.52–1.34)	1.60 (0.81–3.17)	1	0.84 (0.41–1.73)	1.64 (0.87–3.06)	1.87∗ (1.01–3.47)	4.70

^#^The adjusted OR was calculated by binary logistic regression models with adjustment for age, gender, occupation, education, family monthly income, family history of diabetes, alcohol consumption, and exercise.

*P < 0.05.
